# Does artificial intelligence enhance physician interpretation of optical coherence tomography: insights from eye tracking

**DOI:** 10.3389/fcvm.2023.1283338

**Published:** 2023-12-08

**Authors:** Giacomo Maria Cioffi, Natalia Pinilla-Echeverri, Tej Sheth, Matthew Gary Sibbald

**Affiliations:** Division of Cardiology, Hamilton General Hospital, Hamilton Health Sciences, McMaster University, Hamilton, ON, Canada

**Keywords:** optical coherence tomography, artificial intelligence, eye tracking, intracoronary imaging, OCT, Ultreon, AptiVue

## Abstract

**Background and objectives:**

The adoption of optical coherence tomography (OCT) in percutaneous coronary intervention (PCI) is limited by need for real-time image interpretation expertise. Artificial intelligence (AI)-assisted Ultreon™ 2.0 software could address this barrier. We used eye tracking to understand how these software changes impact viewing efficiency and accuracy.

**Methods:**

Eighteen interventional cardiologists and fellows at McMaster University, Canada, were included in the study and categorized as experienced or inexperienced based on lifetime OCT use. They were tasked with reviewing OCT images from both Ultreon™ 2.0 and AptiVue™ software platforms while their eye movements were recorded. Key metrics, such as time to first fixation on the area of interest, total task time, dwell time (time spent on the area of interest as a proportion of total task time), and interpretation accuracy, were evaluated using a mixed multivariate model.

**Results:**

Physicians exhibited improved viewing efficiency with Ultreon™ 2.0, characterized by reduced time to first fixation (Ultreon™ 0.9 s vs. AptiVue™ 1.6 s, *p* = 0.007), reduced total task time (Ultreon™ 10.2 s vs. AptiVue™ 12.6 s, *p* = 0.006), and increased dwell time in the area of interest (Ultreon™ 58% vs. AptiVue™ 41%, *p* < 0.001). These effects were similar for experienced and inexperienced physicians. Accuracy of OCT image interpretation was preserved in both groups, with experienced physicians outperforming inexperienced physicians.

**Discussion:**

Our study demonstrated that AI-enabled Ultreon™ 2.0 software can streamline the image interpretation process and improve viewing efficiency for both inexperienced and experienced physicians. Enhanced viewing efficiency implies reduced cognitive load potentially reducing the barriers for OCT adoption in PCI decision-making.

## Introduction

To employ intracoronary imaging findings in procedural decisions, physicians require the ability to perform real-time image interpretation with speed and accuracy (1). The lack of such proficiency among many interventional cardiologists limits the adoption of optical coherence tomography (OCT) despite its clinical utility (2). The use of artificial intelligence (AI) to automate substantial portions of the image interpretation task may improve physician experience with imaging (3–6). The image review software Ultreon™ 2.0 improves on AptiVue™ by using AI to detect calcium and external elastic lamina (EEL), reducing the need for physician identification of these structures. In addition, a simplified interface is used that toggles different representations of the vessel lumen rather than simultaneously showing multiple representations, thereby reducing the cognitive load on the physician (7, 8). The goal of these changes is to streamline the interpretation process for less experienced physicians.

Eye-tracking studies are a way to gain insight into the ease with which physicians extract information from these two software platforms. Eye tracking has been used to investigate decision-making in other fields such as psychology, neuroscience, marketing, human–computer interaction, and medicine (9–18). It provides detailed information on where and how long participants focus on different elements of a visual scene, whether their searching is strategic and whether their gaze coincides with the location of the pertinent information of the visual scene (17, 19).

In this study, an eye-tracking technology was used to understand how visual changes in the OCT software platform from AptiVue™ to Ultreon™ 2.0 impact the viewing efficiency and accuracy of physicians using OCT for percutaneous coronary intervention (PCI) decision-making. We compared experienced and inexperienced physicians to explore the impact differed among who were physicians who experienced vs. inexperienced with the AptiVue™ platform.

## Methods

We conducted a descriptive study of eye-tracking patterns of interventional cardiologists reviewing OCT console images from both Ultreon™ 2.0 and AptiVue™ software platforms.

### Participants

Interventional cardiologists and fellows in training were recruited from a tertiary center in Canada. Operators were considered inexperienced if they self-reported performing less than 50 OCT studies as primary operator with the AptiVue™ platform. Written informed consent was obtained from all participants.

### Imaging materials and interpretation tasks

**Figure 1:**
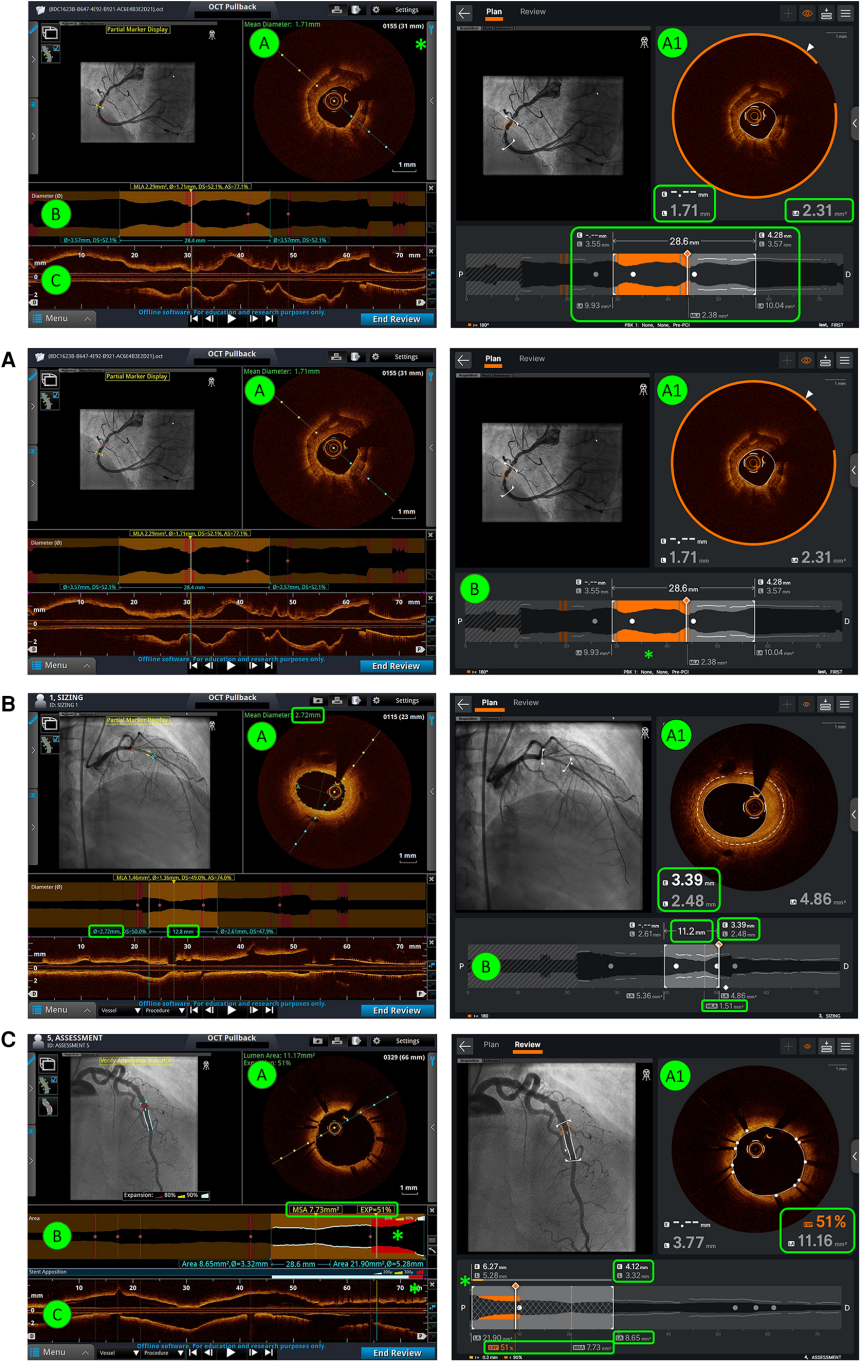
Comparison of AptiVue™ and Ultreon™ 2.0 interfaces. In the AptiVue™ software, panel A provides a cross-sectional image with the possibility of utilizing measurement tools inside the curtain menu (*) (length, area, thickness, angle) and a visual estimation of the mean diameter (green text). In Ultreon™ 2.0, all information is at a glimpse in panel A1. AI-plaque morphology recognizes the calcium burden at a given threshold (orange arc), mean EEL diameter (dashed line - E), lumen diameter (L), and mean lumen area (LA) (green-highlighted boxes). B and C are fused together in Ultreon™ 2.0. This provides several information (highlighted green box): in orange the calcium-plaque burden above the preselected threshold, lesion length, EEL diameter, lumen diameter and LA at the proximal and distal references, and MLA. (**A**) Morphology assessment. In comparison with AptiVue™, Ultreon™ 2.0 presents AI-plaque recognition (A1) with orange band recognizing calcium. This is also presented in orange bands in the longitudinal/lumen profile (B) where the calcium is detected (*) based on the preselected threshold (in this case ≥180°). (**B**) Stent sizing. In comparison with AptiVue™, Ultreon™ 2.0 presents AI recognition of EEL (dashed line - E) which is displayed in the cross-sectional area (A1) and the lumen/longitudinal section (B). In addition, L and LA are displayed at the cross-sectional area (A1) and at the proximal and distal references together with the selected length (B). MLA is also shown (B). (**C**) Stent assessment. In comparison with AptiVue™, Ultreon™ 2.0 fuses panels B and C and provides expansion, MSA (highlighted box), and apposition (*) bars combined in one picture. Expansion at the selected frame and LA values is also shown in panel A1. In addition, L and LA are displayed at the cross-sectional area (A) and at the proximal and distal references.

Six static images were selected for each of three OCT imaging tasks, namely, (1) calcium identification, (2) stent sizing, and (3) stent assessment. Two versions of each static image were prepared, one using Ultreon™ 2.0 and the other using AptiVue™. Two counterbalanced sets of 18 images were constructed such that each set contained six images of each task, half on Ultreon™ 2.0 and half on AptiVue™, but only one version of each image is required in each set so that a participant would only see each image once. Example images of morphology assessment, stent sizing, and stent review are shown in Figure 1 contrasting the visual interface of Ultreon™ 2.0 and AptiVue™.

For calcium identification, participants were asked to interpret if the calcium arc was more or less than 180° based on the static image. For stent sizing, participants were required to give the correct stent diameter and length. Stent diameter was based on rounding down from the distal EEL diameter (if available) or rounding up from the distal lumen diameter. Stent length was based on the measurement identified by both software platforms. For stent assessment, participants needed to identify whether any OCT-identified concerns included a composite of (1) significant edge dissection (defined as a 60° arc and more than 3 mm length), (2) malapposition (defined as more than 300 μm from stent struts to the lumen), and (3) underexpansion (defined as less than 90% of the tapered reference) (20). If one or more features were not identified, the answer was deemed incorrect.

### Training

All participants were oriented to both platforms. All inexperienced operators received formal training in the Ultreon™ 2.0 software. This training consisted of a standardized instruction tutorial provided by Abbott Vascular, structured in two separate videos on pre- and post-PCI image review following the MLD MAX algorithm (21).

### Image review and eye tracking

**Figure 2:**
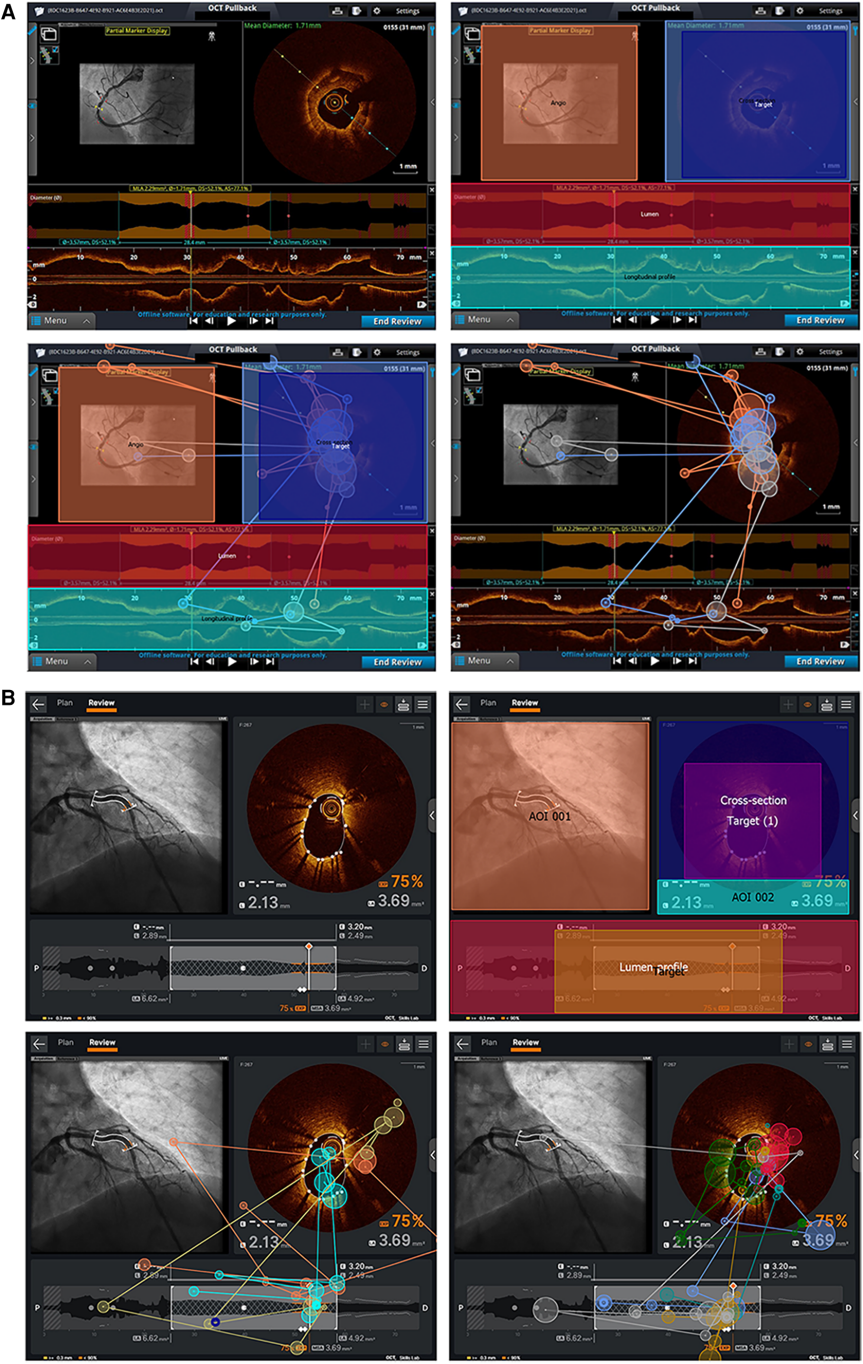
AOI and sample eye–tracking data for Ultreon™ 2.0 and AptiVue™ platforms. (**A**) AOI and target creation for one of the stent result assessment tasks in the Ultreon™ 2.0 group. Right upper picture: AOI is defined by delimiting specific areas/boxes (Target and Target 1) that the software will recognize as areas where gaze/fixation data must be registered. Left and right bottom pictures: Fixation data of inexperienced and experienced operators, respectively, undertaking this stent assessment task without the overlying AOI and target boxes. (**B**) AOI and target creation for one of the calcium assessment tasks in the AptiVue™ group. Left upper picture: The image is imported into the eye–tracking dedicated computer and defined as a task image. Right upper picture: Areas of interest are defined by delimiting specific areas/boxes that the software will recognize for areas where gaze/fixation data must be registered. The “target” box defines the area of interest for the specific analysis. Left bottom picture: Fixation data of experienced operators undertaking this task with overlying AOI and target. Right bottom picture: Fixation patterns of experienced operators without the overlying AOI and target boxes.

Participants were randomized to review one of the two sets of 18 static images. Images were equally distributed between each of the three different tasks and each of the two console types. Participants provided an interpretation while their eye movements were tracked. Eye movements were recorded using an SMI RED 250 Hz eye tracker (SensoMotoric Instruments, Germany), a non-invasive tool which uses a small infrared camera mounted at the bottom of a laptop screen to follow the pupils. Areas of interest (AOI) specific to each task and software system were graphically identified within the static images and verified by two experts (GC and MS). Within each task, the most relevant area of interest (in special cases multiple areas of interest) was determined as the area of interest containing all necessary information to correctly complete the task as shown in Figure 2.

### Outcomes

The primary study outcomes were viewing efficiency and interpretation accuracy. Viewing efficiency was assessed as follows: (a) time to first fixation (time taken for the participant to look at the target area of interest for the prescribed task), (b) total task time (time taken to complete the prescribed task), and (c) dwell time in target defined as the percentage time in target compared with total task time (s). Interpretation accuracy was defined as the proportion of correct responses provided by the participant. Heat maps of eye movements were created to qualitatively compare experienced vs. inexperienced operator eye movements.

### Data analysis

For each participant and task, viewing efficiency and accuracy metrics were averaged and subjected to a mixed multivariate model. Platform type (AptiVue™ vs. Ultreon™ 2.0) and task type (identification of calcium, stent sizing, stent assessment) were treated as within-subject variables. Operator experience (inexperienced vs. experienced) was treated as a between-subject variable. Statistical analysis was performed using SPSS version 26 (IBM, Redmond).

Heat maps were reviewed by three OCT imaging experts (GC, TS, MS), to identify key themes in the viewing patterns using an inductive approach with constant iterative comparisons across platforms, experience levels, and task.

## Results

**Figure 3:**
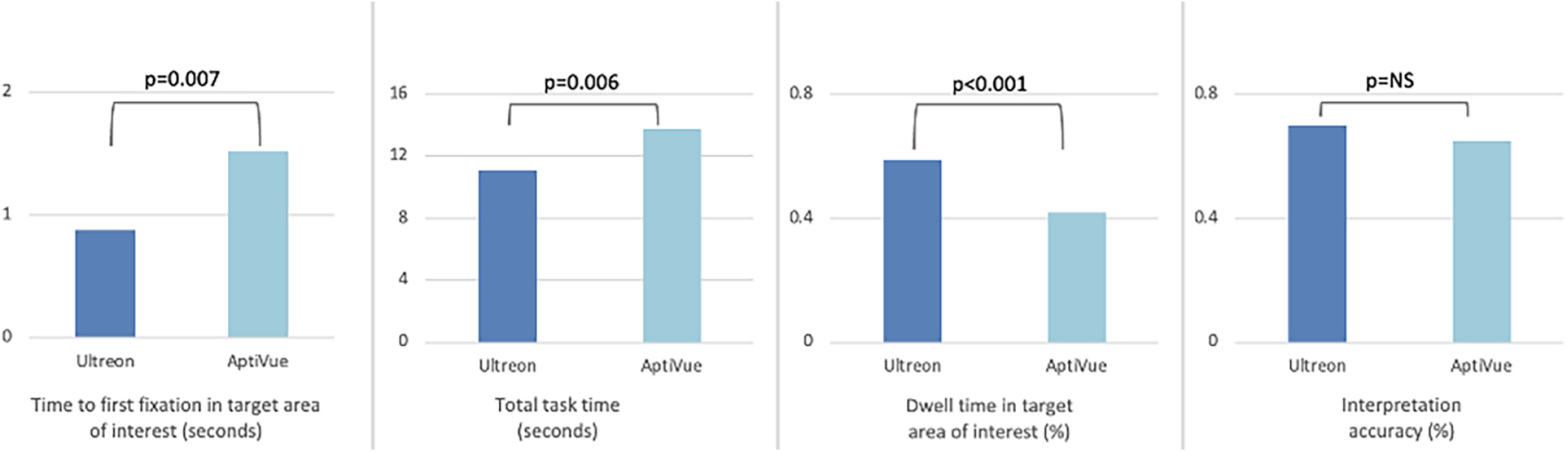
Comparison of viewing efficiency and interpretation accuracy metrics between Ultreon™ 2.0 and AptiVue™ platforms. The use of the Ultreon™ 2.0 platform compared with AptiVue™ while performing three interpretation tasks relevant to percutaneous coronary intervention (quantifying the arc of calcium, choosing a stent size, assessing a stent result) was associated with reduced time to first fixation in the most relevant target area of interest, reduced total task time, increased dwell time in the target area of interest, and preserved interpretation accuracy.

Eighteen physicians participated in the study. Of these, 10 (56%) were practicing interventional cardiologists, and eight (44%) were fellow physicians [two (11%) were women]. Twelve (67%) self-reported performing less than 50 OCTs as first operator and were considered inexperienced. Tables 1 and 2 summarize the results by experience level and task type. Figure 3 compares average viewing efficiency and interpretation accuracy between the Ultreon™ 2.0 and AptiVue™ platforms.

**Table 1:** Viewing efficiency stratified by the participant experience level.

	Inexperienced participants		Experienced participants		All participants	
	AptiVue	Ultreon	AptiVue	Ultreon	AptiVue	Ultreon
Time to first fixation in target (s)	1.38 ± 2.76	0.91 ± 1.93	1.80 ± 2.77	0.82 ± 1.63	1.52 ± 2.77	0.88 ± 1.83
Total task time (s)	16.0 ± 10.2	12.9 ± 8.2	9.3 ± 5.9	7.6 ± 5.5	13.8 ± 9.5	11.1 ± 7.8
Dwell time in target (%)	46 ± 28	62 ± 27	36 ± 29	53 ± 32	42 ± 29	59 ± 29

**Table 2:** Viewing efficiency results stratified by task.

	Calcium identification		Stent planning		Stent assessment	
	Ultreon	AptiVue	Ultreon	AptiVue	Ultreon	AptiVue
Time to first fixation in target (s)	0.22 ± 1.18	0.59 ± 2.46	1.67 ± 2.35	2.67 ± 2.98	0.74 ± 1.49	1.35 ± 2.47
Total task time (s)	6.2 ± 4.2	8.7 ± 7.3	13.5 ± 5.8	18.3 ± 10.5	13.8 ± 9.8	14.3 ± 8
Dwell time in target (%)	80 ± 26	66 ± 27	46 ± 28	25 ± 19	52 ± 23	37 ± 22

### Viewing efficiency

Time to first fixation (TFF) in target was low, averaging 1.2 s (95% CI 1.0–1.5 s). In a mixed multivariate analysis, Ultreon™ 2.0 TFF was 0.9 s (95% CI 0.5–1.2 s) vs. 1.6 s for AptiVue™ (95% CI 1.2–2.0 s, *p* = 0.007) (Table 3). The reduced TFF using the Ultreon™ 2.0 platform was present for both inexperienced and experienced operators, 0.9 ± 1.9 vs. 1.4 ± 2.8 s and 0.8 ± 1.6 s vs. 1.8 ± 2.8, respectively. The effect was also consistent across tasks with TFF significantly reduced for the calcium assessment (0.2 vs. 0.6 s), stent planning (1.7 vs. 2.7 s), and stent assessment tasks (0.7 vs. 1.3 s). Interactions between software platform and experience level or task type were not significant.

**Table 3:** Mixed multivariate models for viewing efficiency metrics and accuracy.

	Time to fixation on target		Task time		Dwell time in target		Accuracy	
	*F*	*p*	*F*	*p*	*F*	*p*	*F*	*p*
Intercept	82.64	<0.001	690.1	<0.001	1,229.166	<0.001	805.322	<0.001
Platform	7.451	0.007	7.6	0.006	36.428	<0.001	0.206	0.651
Task	16.484	<0.001	39.0	<0.001	73.010	<0.001	12.034	<0.001
Experience	0.283	0.595	48.4	<0.001	11.510	<0.001	32.626	<0.001
Platform × task	0.513	0.599	2.3	0.101	0.621	0.538	0.197	0.821
Platform × experience	0.856	0.356	0.6	0.435	0.001	0.971	3.625	0.058

Total task time (TTT) averaged 11.4 s (95% CI 10.6–12.3 s). TTT was lowered using the Ultreon™ 2.0 platform (10.2 s, 95% CI 9.0–11.5, vs. 12.6 s, 95% CI 11.4–13.9 for AptiVue™, *p* = 0.006) both among inexperienced (12.9 ± 8.2 vs. 16.0 ± 10.2 s) and experienced (7.6 ± 5.5 vs. 9.3 ± 5.9 s) operators. TTT also exhibited a significant reduction across the different tasks (6.2 vs. 8.7 s for the calcium assessment, 13.5 vs. 18.3 s for the stent planning, and 13.8 vs. 14.3 s for the stent assessment).

Dwell time (DT) in the target area of interest averaged 49% (95% CI 47%–52%). It was significantly increased using the Ultreon™ 2.0 platform (58%, 95% CI 54%–67%, vs. 41%, 95% CI 37%–44%, *p* < 0.001). DT also significantly varied across experience and task (both *p* < 0.001). However, the interaction variables with the platform for both experience and task were not significant.

### Interpretation accuracy

Overall interpretation accuracy of the OCT images was 73% (95% CI 0.67–0.78). In a mixed multivariate analysis, the software platform did not affect interpretation accuracy (Ultreon™ 74%, 95% CI 67%–81%, vs. AptiVue™ 71%, 95% CI 64%–78%, Table 3). However, experienced participants outperformed inexperienced participants (87%, 95% CI 79%–95%, vs. 58%, 95% CI 52%–64%, *p* < 0.001). Interpretation accuracy also varied across task type (calcium identification 82%, 95% CI 73%–90%; stent sizing 56%, 95% CI 47%–64%; stent assessment 80%, 95% CI 72%–88%, *p* < 0.001). Interactions between the software platform and experience level or task type were not significant.

### Heat maps

**Figure 4:**
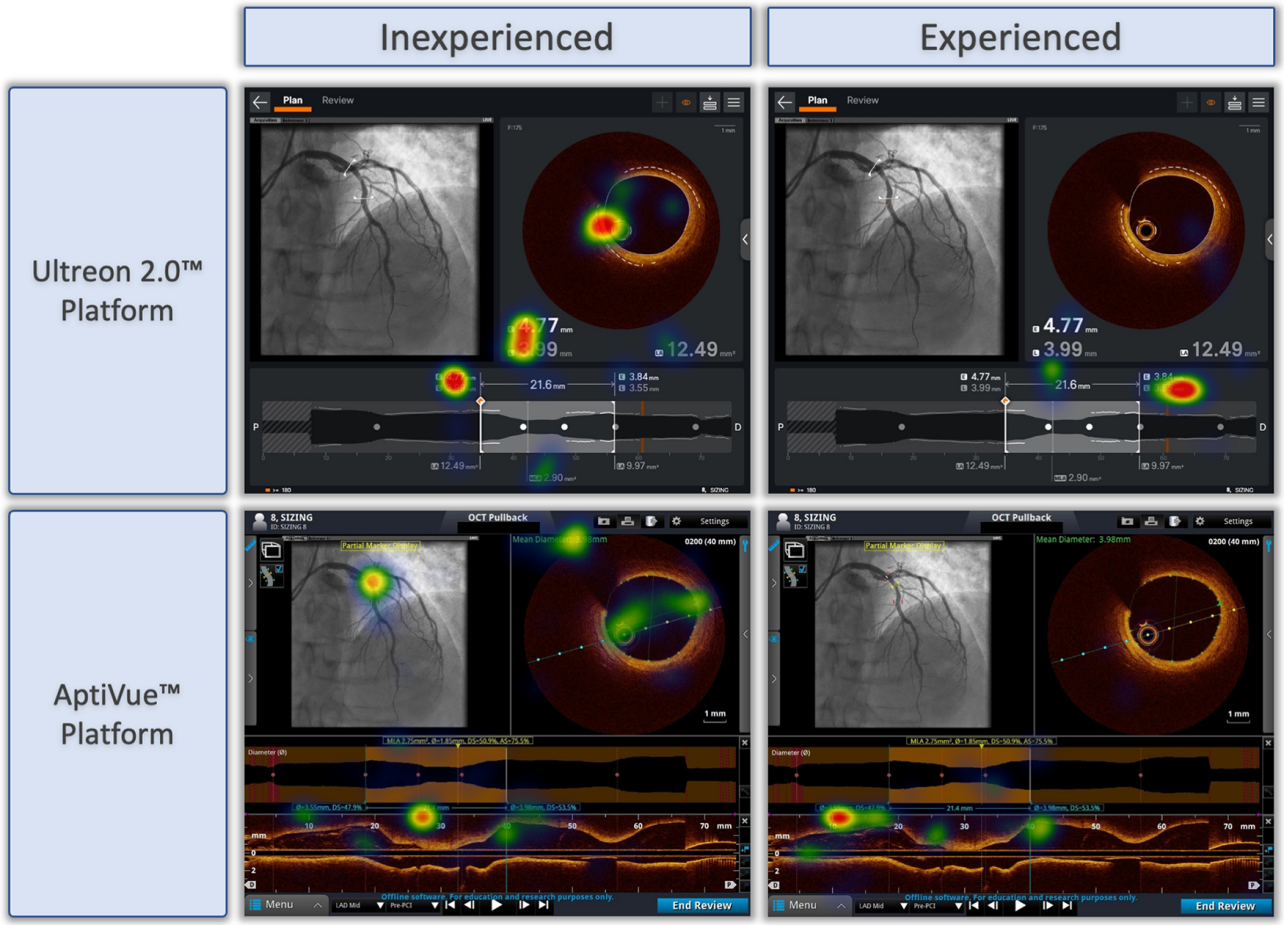
Representative heat maps of eye tracking during OCT-guided stent sizing task with Ultreon™ and AptiVue™ platforms. Eye-tracking heat maps which identify areas of fixation, with warm colors reflecting longer duration of gaze, and colder colors less duration of gaze. Representative samples of inexperienced and experienced participants deciding on stent size based on OCT imaging using the Ultreon™ 2.0 and AptiVue™ platforms.

Qualitative review of heat maps showed fewer areas of focus in Ultreon™ 2.0 vs. AptiVue™ platforms and fewer areas of focus among experienced vs. inexperienced participants. A representative sample of heat maps for the stent sizing task is shown in Figure 4. Both experienced and inexperienced participants focused on key numbers in the Ultreon™ 2.0 platform but had more areas of focus in the cross section and longitudinal view in the AptiVue™ platform.

## Discussion

This is the first study to use eye tracking to explore physician interpretation of intravascular imaging using an AI-enhanced software. Two key findings are reported: (1) Ultreon™ 2.0 improved the viewing efficiency of both inexperienced and experienced physicians compared with AptiVue™, and (2) physicians experienced in intracoronary imaging using the AptiVue™ platform retained their expertise and accuracy in the Ultreon™ 2.0 platform.

Eye tracking has been used to evaluate physician performance in non-invasive imaging interpretation tasks (13, 15). Much of the literature comes from radiology where expertise is associated with decreased time on task and quicker focus on relevant areas of interest (13). Both metrics (TTT and TFF) were better among experienced vs. inexperienced physicians in this study. More importantly, the potential impact of comprehensively integrating AI into the image interpretation process was also assessed in this study. The Ultreon™ 2.0 software was associated with improvements in TTT, TFF, and DT for both inexperienced and experienced physicians suggesting that the streamlined interface and AI integration made both groups more proficiently behave in their viewing patterns. Thus, the phenomenon of “expertise reversal,” in which experienced physicians who have already accustomed to the greater amount of visual information that the AptiVue™ software provides might have had their performance worse, did not occur (22).

AI-enabled systems, such as Ultreon™ 2.0, hold a promise to improve time-sensitive decisions as part of a broader approach of collaboration between physicians and AI (23). Importantly, whether physicians can leverage AI involves a host of human and technological factors. In this study, physicians leveraged the AI-enhanced OCT data with minimal training regardless of their levels of expertise. This ease of use relates to the intrinsic interpretability of the AI enhancements which are displayed as a visual overlay on top of raw cross-sectional imaging data, a concept termed explainability within the AI literature (24, 25). Many motivations for incorporating AI explainability into the interface design including a desire to help physicians justify AI decisions, correct errors, and build trust through knowing why the AI output was produced are noted (26). This approach may account for the successes of integrating AI within the Ultreon™ 2.0 platform in contrast to mixed success in integrating AI in other areas (3).

The enhanced viewing efficiency noted using Ultreon™ 2.0 holds relevance for clinical practice and the cognitive load of physicians seeking to use OCT datasets in planning PCI. Cognitive load theory suggests that effective learning and decision-making processes are limited by the amount of information that working memory can process at any given time (7). The high-resolution, cross-sectional dataset from OCT has a wealth of information to guide PCI. However, to take a full advantage of this dataset, physicians must process and interpret this additional information under the time pressures of clinical care, a scenario that may test the limits of their working memory (8). By standardizing the OCT workflow (21), and using AI-enhanced software to focus clinician attention on this standardized workflow, the use of cognitive load barriers could be reduced. In the LightLab experience, a standardized OCT workflow meaningfully impacted physician PCI decisions and reduced equipment use but increased procedure time by 9 min on average (27). Our eye-tracking findings suggest that Ultreon™ 2.0 may streamline the interpretation process by reducing complex information into manageable chunks, thus aligning with the principles of cognitive load theory.

## Study limitations

Several limitations are worth mentioning. First, our study was conducted using static images that forced the interrogated physician to provide interpretation and answers without the ability to dynamically manipulate the image. This simplifies the task and would potentially underestimate the streamlining provided by Ultreon™ 2.0 compared with AptiVue™. Second, while our participants had diverse training and experience with OCT, all were recruited from a single center.

## Conclusions

Using OCT to make PCI decisions, physicians had improved the viewing efficiency with preserved accuracy using an AI-enabled, visually streamlined Ultreon™ 2.0 software compared with the AptiVue™ platform.

## Data Availability

The raw data supporting the conclusions of this article will be made available by the authors, without undue reservation.
